# Statistical Discrimination of Urinary Steroid Biomarkers in the Athlete Biological Passport: A Novel Approach to an Abnormal Steroid Profile Score (ASPS)

**DOI:** 10.1002/dta.70054

**Published:** 2026-03-05

**Authors:** James G. Hopker, Jim E. Griffin, Matthew N. Fedoruk, Laura A. Lewis

**Affiliations:** ^1^ School of Sport and Exercise Sciences University of Kent Canterbury UK; ^2^ Department of Statistical Science University College London London UK; ^3^ United States Anti‐Doping Agency Colorado Springs Colorado USA

**Keywords:** antidoping, athlete biological passport, Bayesian adaptive model, multivariate analysis, testosterone

## Abstract

The steroidal module of the Athlete Biological Passport (ABP) longitudinally monitors five ratios between urinary concentrations of endogenous anabolic and androgenic steroids. Even though it has improved detection of testosterone doping, the interpretation of data from multiple discrete biomarkers is complex. This study sought to create a single score to identify doping rather than relying on the interpretation of each parameter alone. A Bayesian model was used to define an *ABP sequence probability* for each biomarker to assess the extremity of a measurement relative to the expected levels from ABP. This was used to discriminate between doped and presumed clean individuals based upon pattern classification of biomarkers using classification algorithms. Data were obtained from laboratory‐controlled experimental studies as well as routine doping control tests. A laboratory model (where classifier is trained using the laboratory‐controlled data only) and a mixed model (where classifier is trained on combined laboratory‐controlled and doping control data) were developed and tested on the doping control data. Logistical regression was seen to have the best classification performance across the methods used, with the Abnormal Steroid Profile Score (ASPS) representing the estimated probability from the logistical regression model. Classifier performance produced an AUC of 0.67 and 0.75 when trained on the laboratory model and the mixed model, respectively, with T/E and 5α‐Diol/5β‐Diol representing the main biomarkers driving the ASPS. These findings demonstrate that the ASPS can discriminate between the doping status of individuals, even if a mixture of steroids, administration methods and doses are used.

## Graphical abstract

The study developed a single Abnormal Steroid Profile Score (ASPS) to improve interpretation of the Athlete Biological Passport steroid module. Using Bayesian modelling and logistic regression, biomarker patterns from laboratory and doping control data successfully discriminated between doped and clean individuals, with improved classification performance when data sources were combined.

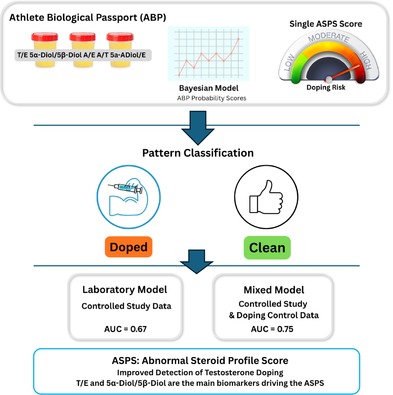

## 1 Introduction

Athletes dope using performance‐enhancing drugs (PEDs) to artificially increase their sport performance. Many substances and methods encompass the WADA Prohibited List, including, but not limited to, blood doping using erythropoietin stimulating agents or a blood transfusion or steroid doping via exogenous or pseudo‐endogenous anabolic androgens. From an antidoping perspective, some of these methods and hundreds of substances and their metabolites can be detected via direct antidoping tests, but due to some synthetic prohibited substances being almost indistinguishable from endogenous hormones and proteins, indirect tests are also used to monitor biomarkers of blood and/or urinary and blood steroid metabolites on a longitudinal basis for the identification of potential doping cases through the Athlete Biological Passport (ABP).

The ABP uses a Bayesian adaptive model to generate population informed but individual‐based upper and lower limits for each athlete, which adapt over time as more test samples are added and allow the setting of predicted ranges of biomarkers for each individual. Biomarker values that fall outside of the predicted individual ranges can then be flagged for follow‐up evaluation by Athlete Passport Management Units [1]. Expert analysis aims to examine the likelihood that the atypical nature of the observed biomarker value is due to doping, normal physiological variability explained by confounding factors or an acute or chronic medical condition. Although the ABP has greatly increased the potential for detection and deterrence, there remains great interest in examining the interplay and possible associations between multiple parameters in hopes that increases in ABP sensitivity or specificity may be observed [2, 3, 4, 5, 6].

The steroidal module of the ABP was introduced to longitudinally monitor the concentration of endogenous steroids and their ratios over time [1, 7]. Specifically, urinary steroidal metabolite concentrations of testosterone (T), epitestosterone (E), androsterone (A), etiocholanolone (Etio), 5α‐androstane‐3α,17β‐diol (5α‐ADiol) and 5β‐androstane‐3α,17β‐diol (5β‐ADiol) are used to generate biomarker ratios of T/E, A/T, A/Etio, 5α‐ADiol/5β‐ADiol and 5α‐ADiol/E [8]. However, due to the multiple analytes that are measured, the subsequent interpretation of data obtained from antidoping control tests is complex. As such, analysis requires the integration of multiple discrete biomarker measurements in the context of differentiating normal biological profiles from abnormal ones, likely caused by doping.

Recently, Eleftheriou et al. [6] have presented a multivariate extension of the steroid module of the ABP that jointly models the biomarkers to address these complexities. Their research demonstrates improved detection of doping incidence compared to the existing univariate adaptive model. Deliu and Liseo [9] have also recently examined a similar multivariate approach to doping detection using haemoglobin concentration and OFF‐score biomarkers within the haematological module of the ABP. However, more data are required to assess the statistical performance of their method.

Within the haematological module of the ABP, the abnormal blood profile score (ABPS) was developed [2] to identify blood doping via the combination of seven haematological markers (reticulocyte percentage, haemoglobin concentration, haematocrit, red blood cell count, mean corpuscular volume, mean corpuscular haemoglobin and mean corpuscular haemoglobin concentration) into a single ABPS Score. The aim of the ABPS [2] is to discriminate between doped and nondoped individuals based upon statistical pattern classification of the biomarkers listed above. Scores between 0 and 1 are derived with the higher score, suggesting an increased probability of doping, with a score of 1 being found in 1 in 1000 clean male athletes. Indeed, the ABPS is based upon two different classification techniques, a naive Bayesian classifier and support vector machine learning with output scores being combined using ensemble averaging to generate the final score. However, no equivalent to the ABPS currently exists for the urinary steroidal module of the ABP.

Therefore, rather than taking a generative approach to understand biomarker interaction [6, 9], we propose a discriminative approach based upon an abnormal steroid profile score (ASPS), which builds features from the biomarkers for doping classification. This approach allows us to more easily allow for complex relationships between biomarkers and changes in the profile over time without making restrictive distributional assumptions. Accordingly, the aim of this research is to generate an ASPS that can be used to discriminate between doped and clean athletes in the context of testosterone (T) doping. As part of this work, we develop a multivariate statistical model to define a sequence probability for each biomarker of the Steroidal ABP and subsequently test a range of statistical and machine learning discriminative algorithms to classify indirect markers of altered steroid metabolites. We combine data from laboratory‐controlled longitudinal experimental studies [10, 11] involving transdermal and intramuscular testosterone administrations, as well as a data set obtained from routine doping control tests. We assess the utility of the ASPS to generate risk scores for longitudinal steroid profiles as well as its predictive performance using out‐of‐sample statistical testing.

## 2 Data Sources

### 2.1 Laboratory Controlled Dataset

We used data previously collected in two different laboratory‐controlled experimental studies. In study 1^10^, 15 participants were administered T using transdermal patches (1 g of a 4% T gel for 2 weeks) and two intramuscular injections (200 mg T enanthate) 2 weeks apart. During transdermal patch administration, urine samples were collected twice a week during the ‘doping’ phase and for five consecutive days after the last application (washout). Three months after the transdermal administration, participants received the intramuscular injection administration. A total of 40 urine samples were collected during the period of administration and subsequent washout. Data were analysed per participant as a change of measured biomarkers concentration values across the doping regime.

In study 2^11^, 15 healthy male volunteers self‐administered either a 50‐, 75‐ or100‐mg testosterone dose weekly for 4 weeks using a Xyosted testosterone enanthate single use administration device (Antares Pharma, Ewing, NJ, United States). A total of 24 urine samples were collected 3 days prior to administration, 3 days per week during the administration and over a 13‐day period following the last administration. Data were analysed per participant as a change of measured biomarkers concentration values collected following each self‐administered injection.

### 2.2 Doping Control Dataset

The data set consisted of steroid ratios (T/E, A/Etio, 5α‐ADiol/5β‐ADiol and 5α‐ADiol/E) obtained from urinary steroid measurements performed on routine doping control (urine) samples from 13,646 athletes by the United States Anti‐Doping Agency, of which 495 were later sanctioned for doping offences (13,151 athletes remained unsanctioned at the time data were obtained). The number of doping control samples differed between athletes, ranging from 1 to 76 for sanctioned athletes (median = 4 samples and mean = 9 samples) and between 1 and 72 for unsanctioned athletes (median = 2 samples and mean = 4 samples). The number of adverse analytical findings also differed between the sanctioned (between 1 and 20 adverse findings: median = 1 sample and mean = 1.139 samples), with 82 athletes having more than one adverse analytical finding within their data. For the purposes of risk quantification, we restricted the analysis to athletes with more than three doping control measurements. This resulted in 3905 athletes within the sample, of which 286 had an ADRV.

## 3 Methods

Following institutional ethical approval, we analysed the ratios of biomarkers: T/E, A/Etio, 5α‐ADiol/5β‐ADiol, 5α‐ADiol/E and A/T.

### 3.1 Linear Mixed Model for Laboratory Only Data

To understand the relationship between doping regime and the level of each biomarker, we initially analysed the two laboratory experimental studies using a linear mixed effects model. For each biomarker, a linear mixed effects model was fitted to the logarithm of the biomarker with the change from baseline (of the logarithm) used as the response. Each model included an individual‐specific intercept (which adjusts for heterogeneity in the participants) and fixed effects for the different administration methods and doses.

### 3.2 Classifiers Trained on Laboratory Only Data

We initially followed the approach taken by Sottas et al. [2] by training models on the laboratory data only. We classified samples as either nondoped (corresponding to the baseline measurements) or doped (corresponding to the other measurements). The data set includes different levels of doping, and we investigated whether classifiers trained using higher levels of doping were better or worse for out‐of‐sample prediction in both the laboratory data set and the doping control data set. We used six types of doping, which range from higher levels of doping (Type A) to all doping types (Type F). The six types were as follows:

1. Type A (high‐dose 200‐mg intramuscular injections)—Intramusculars 1 and 2.
2. Type B (high‐dose intramuscular injection)—Intramusculars 1 and 2 and 100‐mg injections.
3. Type C (high‐ and low‐dose intramuscular injections)—Intramusculars 1 and 2 and 75‐ and 100‐mg injections.
4. Type D (low‐dose intramuscular injection and dermal patch)—50‐ and 75‐mg injections and transdermal.
5. Type E (intramuscular injections and dermal patch)—Intramusculars 1 and 2; 50‐, 75‐ and 100‐mg injections; and transdermal.
6. Type F—all nonbaseline samples.

We considered four machine learning classifiers: logistic regression, linear discriminant analysis, naive Bayes and quadratic discriminant analysis (QDA). The analysis was performed using the mlr R package [12].

We also consider two sets of features:

- The original data set: This data set includes the measurements on all biomarkers. We define this to be $y_{i,k,t}$ for the *k*th biomarker of the *i*th athlete at the *t*th sampling occasion.
- ABP sequence probability (ABP‐SP): Steroid biomarker levels vary both within and between individuals. This variation is addressed in doping risk quantification using the ABP and its underlying Bayesian model. This model generates a posterior predictive distribution for each biomarker and individual at each sampling occasion. This distribution for the *i*th athlete and *k*th biomarker at the *t*th sampling occasion is represented by $F_{i,k,t}$. The distribution measures the extremity of a biomarker relative to plausible levels determined by the ABP and is used to generate plausible biomarker limits for each biomarker and athlete at each sampling occasion based on an athlete's previous observations. To create a feature, we define an ABP‐SP, which is a transformation of the biomarkers defined by $z_{i,k,t}=F_{i,k,t}\left(y_{i,k,t}\right)$. A probability close to 0 indicates that the measurement is much lower than expected, and close to 1 indicates that the measurement is much higher than expected.

The classifiers are compared using the Area under the ROC curve (AUC) metric to understand how well methods discriminate between doped and nondoped samples. This measure is widely used to compare predictive performance in binary regression problems and allows us to compare performance without choosing a threshold for the risk score to classify an athlete as doping. The AUC takes values between 0 and 1, with a larger value indicating that a method is better able to discriminate between the two groups of athletes. An AUC score of 1 shows perfect discrimination, whereas a score of 0.5 is equivalent to guessing.

We use two setups to understand different aspects of predictive performance of different classifiers trained using data sets constructed from the different Types and features. These are as follows:

1. Leave one out cross‐validation applied to the laboratory‐controlled sample data set only
2. Using the doping control sample data set is as follows: a validation sample data set. The classifier is used to generate a risk score for each observation in the doping control data set and the largest risk score is used to calculate the AUC metric. This corresponds to a setting where the risk score is calculated on each sampling occasion and an athlete is flagged as doping if they have at least one risk score over a prespecified threshold.

### 3.3 Classifiers Trained on Both the Laboratory and the Doping Control Data (Mixed Data)

We now consider using both the laboratory and doping control sample data to develop a method to quantify risk. In this data set, doping status is unknown, but we can mark an athlete as doping if they have an ADRV during the sample period. However, unlike the laboratory data set, we have a doping marker for each athlete rather than each sampling occasion. This led us to develop a classification method which uses features based on all measured biomarkers for an athlete rather than measured on a single sampling occasion. Our approach was to develop simple, interpretable summaries encompassing *all* observations for a participant. Note that the samples labelled as doped also include wash‐out periods when participants were not actively doping. We define the features in the following way (where a measurement can refer to either a biomarker or ABP‐SP):

- The sample mean of each measurement (5 in total); for example, the sample mean of 5α‐ADiol/E is denoted by Mean(5α‐ADiol/E).
- The range (maximum value minus minimum values) of each measurement (five in total); for example, the range of T/E is denoted by Range(T/E).
- The range of the first difference (the differences between *t*th and *t1*th observation) for each measurement (five in total); for example, the range of the first difference of 5α‐ADiol/E is denoted by Range(Δ5α‐ADiol/E).
- The range of the second differences (the first differences of the first differences) for each measurement (5 in total); for example, the range of the second difference of A/Etio is denoted by Range(Δ^2^A/Etio)
- The sample covariance between pairs of measures (10 in total); for example, the sample covariance between 5α‐ADiol/Etio and 5α‐ADiol/E is denoted by Cov(5α‐ADiol/Etio, 5α‐ADiol/E).
- The sample covariances between pairs of first differences of measurement (10 in total); for example, the sample covariance between the first differences of 5α‐ADiol/5β‐ADiol and 5α‐ADiol/E is denoted by Cov(Δ5α‐ADiol/5β‐ADiol, Δ5α‐ADiol/E).

The sample mean and the range measure the level and spread of each measurement. The range of the first and second differences measures the variability in the rates of increase or decrease for each measurement and so can detect rapid changes in measurement levels. The sample covariance of pairs of measurements characterizes how strongly two measurements move together, and the sample covariances of the first differences characterize how strongly increases and decreases in the measurements are related. In addition, we include both the feature and the square of the feature. This leads to 400 features.

We applied nine machine learning classifiers: logistic regression, linear discriminant analysis (LDA), AdaBoost [13], Random Forest [14], Conditional Forest [15], Boosted Logistic Regression [16], Mixture Discriminant Analysis [17], Naive Bayes and Quadratic Discriminant Analysis (QDA). The analysis was performed using the mlr R package [12]. We trained the classifiers using one set of features (either original or ABP‐SP) and with an athlete marked as doped if they have received an ADRV in the doping control or taken part in the laboratory‐controlled trials (where they were experimentally doped). We used the whole trajectories (including all washouts) of the participants in the laboratory studies as we consider that these may be more representative of trajectories of athletes involved in doping. Because the experimental data set includes known dopers, whereas the doping control data set only markers athletes with an ADRV as dopers, we weight athletes differently in the loss function used to train the classifier. The athletes in the laboratory data were given a weight of 20 in the loss function, which reflects that these observations are known dopers, whereas athletes in the doping control data set were given a value of 1. For comparison, we also consider fitting the classifiers using the doping control data only.

Again, the AUC was used as the performance metric. The doping control sample data set is used in training and so cannot be used as a validation sample data set. We therefore use fivefold cross‐validation. The doping control sample data set is divided into *K* folds with the same number of positive ADRV's in each fold (testing only occurs on the doping control data). For each fold, we train using a combined data set of the other folds and the laboratory studies [10, 11] sample data.

## 4 Results and Discussion

### 4.1 Linear Mixed Model Applied to the Laboratory Sample Data Only

The linear mixed model was fitted to each biomarker. The results of Study 1^10^ are shown in Table 1.

**TABLE 1:** Study 1^10^: Analysis shown as estimate with standard errors in brackets (bold shows results that are significant at a 1% level).

	T/E	5α‐ADiol/E	5α‐ADiol/5β‐ADiol	A/Etio	A/T
Transdermal	**0.41 (0.12)**	**0.48 (0.12)**	**0.16 (0.06)**	−0.05 (0.06)	−0.11 (0.08)
Placebo	**0.32 (0.12)**	**0.31 (0.12)**	0.10 (0.05)	−0.02 (0.06)	−0.14 (0.08)
Intramuscular 1	**2.53 (0.12)**	**1.93 (0.12)**	−0.07 (0.05)	−0.02 (0.06)	**−0.33 (0.08)**
Intramuscular 2	**2.67 (0.12)**	**2.10 (0.12)**	−0.08 (0.05)	0.11 (0.06)	**−0.29 (0.08)**

There are clear differences in the response of each biomarker to the different T administration regimes. T/E and 5α‐ADiol/E show significant differences from baseline for both administration types, whereas A/T shows a significant difference for the intramuscular method only. A/Etio shows no significant differences, and 5α‐ADiol/5β‐ADiol shows a small effect for T patch administration.

**FIGURE 1:**
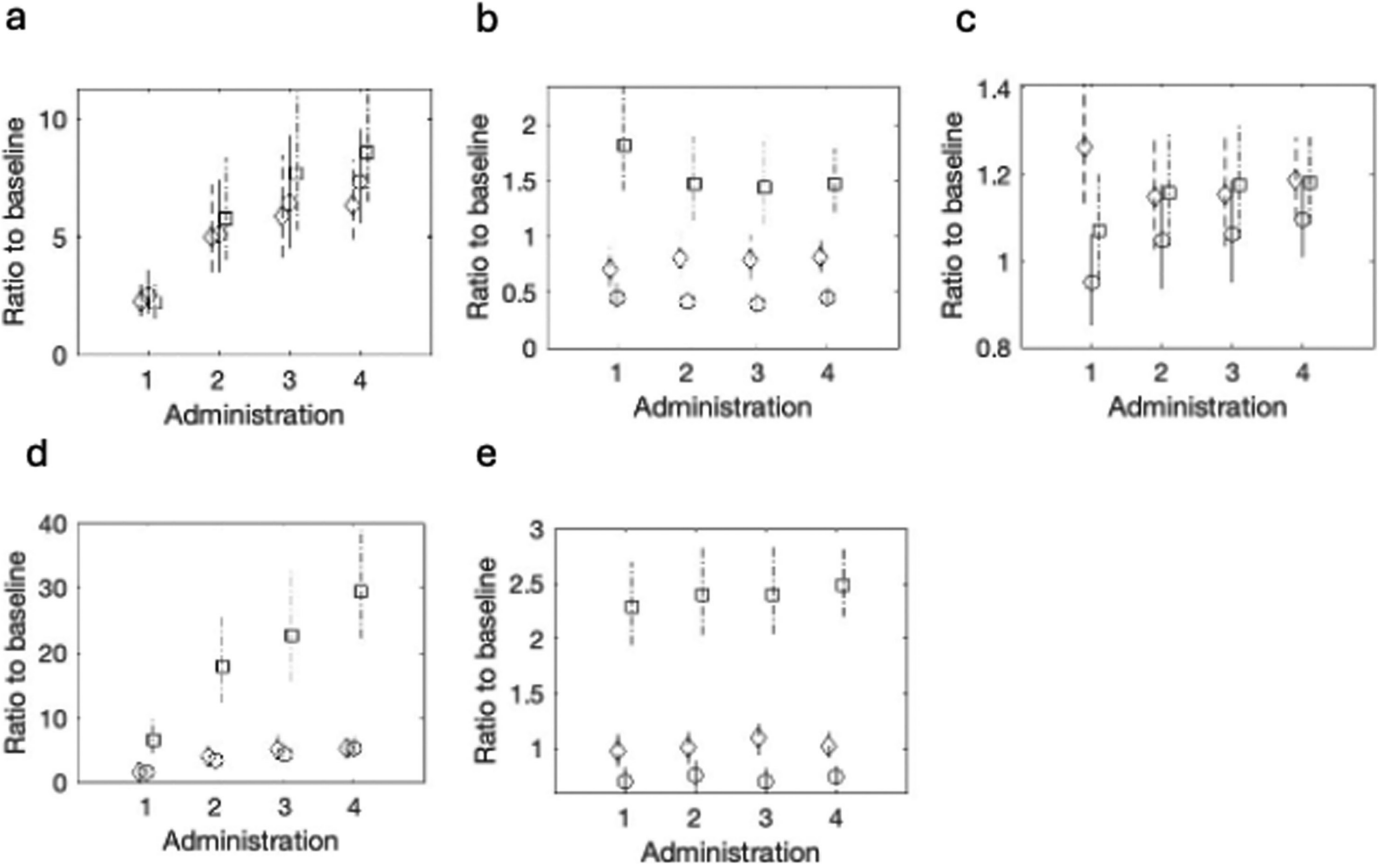
The estimated effect and 95% confidence interval for the log 2 difference of biomarkers (a = TE, b = AT, c = AEtio, d = AE and e = AB) for the four repeated administrations of 50‐mg (circle, solid line), 75‐mg (diamond, dashed line) or 100‐mg (square, dot‐dashed line) T intramuscular injections used in Study 2^11^.

Figure 1 shows the effects of the five biomarkers at the four sampling occasions for the different doses of T intramuscular injection. The results from study 2^11^ show higher levels of T/E compared to baseline, which increase with dose but are not significantly different between dose levels. 5α‐ADiol/5β‐ADiol, 5α‐ADiol/E and A/T show differences from baseline for the 100‐mg dose only. The effect increases with dose for 5α‐ADiol/E but not 5α‐ADiol/5β‐ADiol or A/T. There is limited effect on A/Etio at different doses or dosages.

### 4.2 Analysis of Laboratory Data

The results discriminating between nondoped and different types are shown in Table 2.

**TABLE 2:** Laboratory sample data: The area under the ROC curve (AUC) for various classification algorithms calculated using leave one out cross‐validation applied and trained using a subset of the sample data chosen according to different doses and methods of administration.

Type	Naïve Bayes	Linear discriminant analysis	Logistic regression	Quadratic discriminant analysis
A	0.96	0.96	0.97	0.97
B	0.96	0.96	0.97	0.97
C	0.88	0.90	0.93	0.88
D	0.77	0.75	0.85	0.75
E	0.85	0.82	0.91	0.85
F	0.77	0.70	0.78	0.77

Clearly, the performance of the algorithms deteriorates as the dose applied becomes lower or a dermal application is used. Logistic regression shows very good ability to discriminate in Types A–C and shows a slower deterioration in performance compared to other classification methods. However, all models struggle in the case where we include washout periods (Type F). Although it may be counterintuitive to include washout periods in the sample (because athletes are not actively doping), often athletes will include periods of no drug use within doping regimes.

Below, we compare the performance of classifiers trained on the laboratory sample data using the different type definitions and tested on the doping control sample data set as validation data. The results are shown in Table 3.

**TABLE 3:** Laboratory sample data: The area under the ROC curve (AUC) for various classification algorithms trained on the laboratory only sample data and tested on the doping control sample dataset. Original data are the raw values, and ABP‐SP is the ABP sequence probability.

	Original				ABP‐SP			
	Naïve Bayes	Linear discriminant analysis	Logistic regression	Quadratic discriminant analysis	Naïve Bayes	Linear discriminant analysis	Logistic regression	Quadratic discriminant analysis
A	0.58	0.61	0.61	0.61	0.67	0.57	0.56	0.67
B	0.60	0.61	0.61	0.61	0.67	0.57	0.57	0.66
C	0.61	0.61	0.61	0.61	0.67	0.60	0.57	0.66
D	0.60	0.61	0.61	0.62	0.67	0.57	0.55	0.66
E	0.61	0.61	0.62	0.62	0.67	0.59	0.55	0.67
F	0.61	0.62	0.61	0.62	0.67	0.57	0.55	0.67

Using the original data, we find similar performance across different type definitions and discrimination methods with AUC around 0.61. Better performance is possible with transformed data (i.e., ABP sequence probability) using naive Bayes and QDA methods, leading to an AUC of 0.67. Therefore, when evaluating the robustness of an antidoping measure to discriminate between athletes of different doping statuses, caution should be applied when evaluating the robustness of a laboratory only sample study design (e.g., Alladio et al. [4] and Equey et al. [18]), as it is likely to provide artificially high discriminatory ability compared to a ‘real‐world’ doping control condition due to the high signal and low within and between participant variability. Conversely, in a doping control setting, a much larger within and between athlete variability, as well as doping athletes actively attempting to evade detection, is likely to lead to a much smaller signal to noise ratio. As such, the results shown in Table 3 likely provide a better indication of the performance of the model.

### 4.3 Analysis of the Mixed Data

The results of applying the nine classifiers to both the original and ABP‐SP features use the mixed data and the doping control sample data only.

Table 4 shows results for a range of machine learning methods. First, it is clear that using the ABP sequence probability gives a much better predictive performance than the raw data for all methods considered. Overall, the best performing method was logistic regression using the ABP sequence probability. Therefore, we only consider the logistic regression model within the remainder of this manuscript.

**TABLE 4:** Mixed classification model: The area under the ROC curve (AUC) for different machine learning models fitted using doping control sample data set and a combined doping control and laboratory sample data set.

	Doping control sample data only		Doping control and lab sample data	
Method	Original	ABP SP	Original	ABP SP
Logistic regression	0.61	0.74	0.62	0.73
Linear discriminant analysis	0.63	0.72	0.64	0.71
ADA	0.62	0.70	0.58	0.71
Random forest	0.57	0.70	0.57	0.70
Conditional forest	0.60	0.73	0.60	0.72
Boosted logistic regression	0.61	0.68	0.59	0.65
Mixture discriminant analysis	0.60	0.65	0.59	0.65
Naïve Bayes	0.53	0.65	0.53	0.61
Quadratic discriminant analysis	0.51	0.63	0.55	0.67

Abbreviation: ABP‐SP, ABP sequence probability.

### 4.4 Logistic Regression Classification Model

The validation exercise demonstrated that the logistic regression model had the best classification performance across the competing machine learning methods. We subsequently performed some variable selection to arrive at a (slightly) simpler model with 21 regressors, which also had a slightly larger AUC (0.75).

Table 5 shows the estimated logistic regression coefficients. The model mostly depends on the spreads of variables (or the spreads of the first or second differences of the variables) rather than their levels. The only exception is the level of 5α‐ADiol/E, which has a negative effect on the probability of an ADRV. The risk increases as the range of the biomarkers increases due to either large variability in the profile or extreme values. This relationship is quadratic due to the inclusion of second order terms. Specifically, for A/Etio and 5α‐ADiol/5β‐ADiol, the contribution to the probability of an ADRV is high for large values. Whereas for T/E, the contribution to the probability is highest for low and high T/E scores. The inclusion of first and second differences allows gradients in the profiles to be included in the predictive model, that is, individuals who have large changes in their longitudinal steroidal profiles. As shown in Table 5, the model also includes covariances of the values and values of the first differences of A/Etio, 5α‐ADiol/5β‐ADiol and 5α‐ADiol/E (the covariances involving T/E did not have a strong effect). This allows for large changes in the level or the change in pairs of variables. The ASPS represents the estimated probability from the logistic regression model.

**TABLE 5:** Mixed classification model: The estimated regression coefficients (with standard errors in brackets) of the fitted logistic regression classification model.

Variable	Estimate (SE)	Variable	Estimate (SE)
Intercept	−3.2 (0.1)	Range(Δ5α‐ADiol/5β‐ADiol)	−1.7 (0.2)
Mean (5α‐ADiol/E)	−0.4 (0.1)	Range(Δ5α‐ADiol/E)	−1.7 (0.3)
Cov (A/Etio, 5α‐ADiol/5β‐ADiol)	−1.1 (0.2)	Cov(ΔA/Etio, Δ5α‐ADiol/5β‐ADiol)	0.4 (0.2)
Cov (5α‐ADiol/Etio, 5α‐ADiol/E)	−0.03 (0.11)	Cov(ΔA/Etio, Δ5α‐ADiol/E)	0.07 (0.15)
Cov (5α‐ADiol/5β‐ADiol, 5α‐ADiol/E)	−0.4 (0.2)	Cov(Δ5α‐ADiol/5β‐ADiol, Δ5α‐ADiol/E)	−0.6 (0.4)
Range (T/E)	0.8 (0.2)	Range(Δ^2^A/Etio)	0.6 (0.3)
Range (A/Etio)	0.4 (0.2)	Range(Δ^2^5α‐ADiol/E)	0.9 (0.3)
Range (5α‐ADiol/5β‐ADiol)	2.5 (0.4)	Range(T/E)^2^	0.7 (0.2)
Range (5α‐ADiol/E)	0.2 (0.2)	Range(A/Etio)^2^	0.1 (0.1)
Range (ΔT/E)	−0.9 (0.1)	Range(5α‐ADiol/5β‐ADiol)^2^	−2.6 (0.9)
Range (ΔA/Etio)	−0.9 (0.3)	Range(5α‐ADiol/E)^2^	0.7 (0.2)

### 4.5 Analysis of Individual Biomarkers

The model contains 21 variables, and so it is helpful for the interpretation of the risk to summarise how each biomarker, as well as the interactions of biomarkers, contribute to the ASPS. In this context, each biomarker or the interactions between biomarkers can be viewed as a source of variation in the model. The logistic regression assumes the probability of an athlete with an ADRV is the transformation of a linear combination of the features weighted by the regression coefficients. This allows the model to provide a *risk* score for a source by calculating the contribution to the linear combination (LC) of the features related to that source. We call the contributions for each source: LC‐T/E, LC‐A/Etio, LC‐5α‐ADiol/5β‐ADiol, LC‐5α‐ADiol/E and LC‐Cov. Summing the LC measures will give the same value as calculating the linear combination of the features weighted by the regression coefficients. We only flag LC's larger than 0.8 to give a simple method of deciding, which sources have a large effect on the probability of having an ADRV. Increasing an LC by 0.8 implies an increase of 2.2 in the odds of an athlete doping.

Table 6 shows the flagged LCs for the 15 participants taking part in the laboratory‐controlled experimental study 1^10^ under both T gel and T enanthate conditions, and the 15 participants self‐administered doses of either 100, 75 and 50 mg from study 2^11^. Of the samples analysed, 24/30 have an ASPS above 0.90, including those obtained following both low and high doses of T. The main biomarkers driving this ASPS are T/E (27/30) and 5α‐ADiol/5β‐ADiol (23/30). The LC for 5α‐ADiol/E is over the threshold for 12/30 of participants, which contrasts with the strong effects observed in the analysis of the laboratory only data in Figure 1 and Table 1. This perhaps reflect the consistently of the response of T/E to different dosages and the strong correlation between T/E and 5α‐ADiol/E ( $r^{2}$ = 0.52).

**TABLE 6:** ASPS classification model: The flagged LCs of different sources for 15 participants in the laboratory‐controlled study 1^10^ and following self‐administration of either *100 mg, ^^^75 mg and ^$^50 mg by the 15 participants in study 2^11^.

	Study 1^10^						Study 2^11^					
ID	T/E	A/Etio	5α‐ADiol/5β‐ADiol	5α‐ADiol/E	Cov	ASPS	T/E	A/Etio	5α‐ADiol /5β‐ADiol	5α‐ADiol/E	Cov	ASPS
1	1	0	1	0	1	1.00	1	0	0	0	1	0.90*
2	1	0	1	1	1	1.00	1	0	1	1	1	0.98^^^
3	1	0	1	0	0	0.95	0	0	1	1	1	0.79^$^
4	1	0	1	0	1	0.96	1	0	1	1	0	1.00*
5	1	0	1	1	1	1.00	1	0	0	0	0	0.96^^^
6	1	0	1	0	1	1.00	1	0	0	0	0	0.93^$^
7	1	1	1	0	0	0.95	1	0	1	1	0	0.99*
8	1	0	0	0	0	0.60	0	0	1	1	0	0.65^^^
9	1	0	0	0	0	0.98	1	0	1	0	0	0.42^$^
10	1	1	1	0	1	1.00	1	0	0	0	1	0.74*
11	1	0	1	0	0	0.99	1	0	1	1	0	1.00^^^
12	1	0	1	0	0	0.96	0	0	1	1	1	0.86^$^
13	1	0	1	0	1	1.00	0	0	1	1	1	1.00*
14	1	0	1	0	1	1.00	1	0	1	0	0	0.90^$^
15	1	1	0	1	1	0.98	1	0	1	1	1	1.00^^^

**FIGURE 2:**
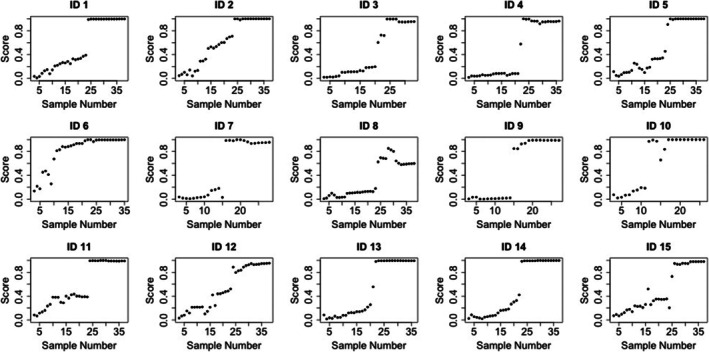
The evolution of the ASPS risk score for participants in laboratory study 1^10^ at baseline (Samples 1–3) following the administration of placebo (Samples 4–14), transdermal patches (1 g of a 4% T gel, Samples 15–25) and two intramuscular injections (200 mg T enanthate, Samples 26–40). Sample number represents the urine sample collection number (total *n* = 40). IDs 7, 9 and 10 have missing placebo administration data and so the total number of data points is less than 40.

**FIGURE 3:**
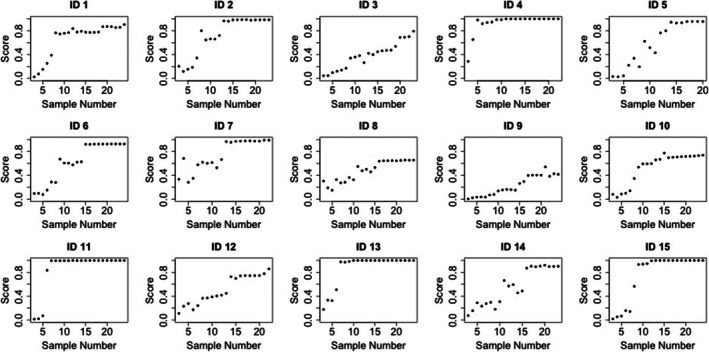
The evolution of the ASPS risk score for participants in laboratory study 2^11^ following self‐administration of: 100 mg ID1, ID4, ID7, ID10 and ID13; 75 mg: ID2, ID5, ID8, ID11 and ID15; and 50 mg: ID3, ID6, ID9, ID12 and ID14.

The results of this study demonstrate that the longitudinal analysis of changes within the ASPS risk score is sensitive to instances of doping. Figures 2 and 3 illustrate the change in ASPS during the laboratory doping period in study 1^10^ and 2^11^, respectively. Even though combined into one ASPS ‘risk score’, the analysis of a range of individual biomarkers within the model has the potential to support a greater understanding of the potential causes of detected atypical athlete profiles. Using multiple biomarkers, rather than relying on individual ratios or concentrations, improves robustness by capturing changes and interactions across the full biomarker set, quantified through their relative contributions to the LC. For example, results suggest a large range of T/E values and a large change in T/E trajectory leads to a higher ASPS. For 5α‐ADiol/5β‐ADiol, the changes in trajectory rather than the range of the concentration level are most important in determining a high ASPS.

Indeed, the previous work of van Renterghem et al. [19] demonstrates that within a longitudinal testing scenario, statistical modelling of multiple biomarkers rather than single ratios or concentrations improves robustness and detection following single low dose 250‐mg dermal DHT and 50‐mg oral DHEA administrations. The same authors later published a follow‐up study [20], in which they demonstrated the efficacy of the same approach using a single oral dose of 40 mg T. However, as both of these studies were designed for a laboratory‐based setting, the sample sizes are small (*n* = 6), and so it is challenging to make inferences to a doping control scenario (with unknown doping regimes used by athletes).

### 4.6 Application of the Abnormal Steroid Profile Score to Doping Control Sample Data

From a practical perspective, the ASPS computes a probability score from the longitudinal behaviour of the ABP steroidal module biomarkers. Specifically, the probability that the change in the biomarker profile would lead to an ADRV. The probability score may therefore be used to help identify the relative risk of the overall athlete profile across the routinely monitored biomarkers, therefore increasing decision‐making ability of the steroidal ABP. Decisions on whether an individual profile should be ‘flagged’ as ‘atypical’ can be made by defining a threshold for the ASPS risk score above which an individual's biomarker trajectory is consider suspicious. We therefore sought to apply the ASPS in a doping control data set to identify its efficacy on a noncontrolled population and the impact of the risk threshold to control the out‐of‐sample false positive rate (FPR). Table 7 shows how the threshold of the ASPS and the true positive rate varies with the false positive rate. We are able to achieve a good true positive rate (TPR) with a low FPR. The minimum ASPS score for the laboratory data is 0.42, which is above the three thresholds in Table 7 below, and so the TPR in the laboratory data is 100% for each threshold. In doping control terms, athletes are likely to utilize a variety of different doping administration regimes, doses and drug combinations in an attempt to circumvent doping control processes. Therefore, the likely sensitivity of doping control processes (i.e., false negative rate) in the field is likely to be lower than observed from laboratory controlled doping studies. To reflect this, within the analysis of the ASPS in the doping control dataset, if a 10% FPR is chosen, it leads to a 37% TPR with 13.5% of athletes being flagged.

**TABLE 7:** Out‐of‐sample false positive rate (FPR) and true positive rate (TPR) for different thresholds of the ASPS. The number and percentage of individuals flagged for a high risk in the doping control sample data are also shown.

Threshold	False positive rate	True positive rate	Number of flagged athletes
0.17	0.05	0.22	291 (7.5%)
0.13	0.10	0.37	528 (13.5%)
0.09	0.20	0.46	1038 (26.6%)

**FIGURE 4:**
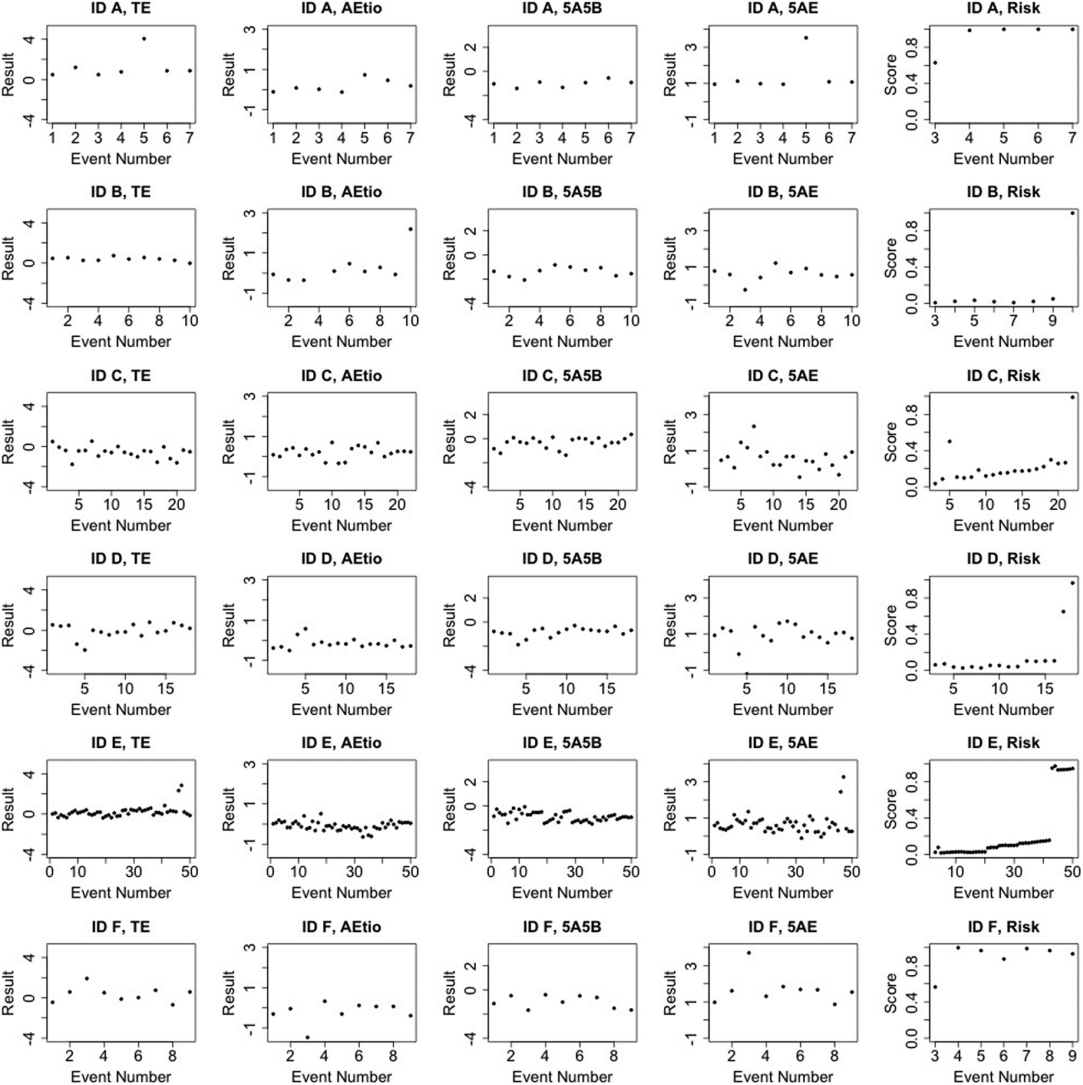
The evolution of the biomarkers and the risk score within the ASPS for athletes with the six highest risk scores from the doping control dataset.

As evidenced in Tables 6 and 8, changes in the T/E ratio appear to be the most responsive biomarker to instances of doping within the model. Results for the six athletes with the highest ASPS within the Doping Control sample data set are shown in Table 8 and Figure 4.

**TABLE 8:** The grouped effects of different measurement types for the six individuals with the highest ASPS in the doping control data set. Athletes with an ADRV are shown in bold.

ID	T/E	A/Etio	5α‐ADiol/5β‐ADiol	5α‐ADiol/E	Cov	ASPS
**A**	1	0	0	0	0	1.00
B	0	1	0	0	1	1.00
C	1	0	0	1	0	0.99
D	1	0	1	1	1	0.97
**E**	1	0	0	1	0	0.95
**F**	1	0	0	0	1	0.94

Athletes A, E and F have S1 ADRVs related to the use of anabolic agents ostarine and methandienone. Due to the historical nature of the doping control dataset, unfortunately, it is not possible to follow up on the suspicious ASPS values with sample reanalysis from athlete IDs B–D. All athletes are flagged by the ASPS for T/E, apart from athlete ID B who is the only one flagged for A/Etio. These results are reflected in the profile of ID B, which is a very flat profile for T/E but demonstrates a large absolute value of A/Etio. All individuals show high variation in their 5α‐ADiol/E profile, but only three are flagged for 5α‐ADiol/E. To assess the potential impact of confounding factors, all samples, including those that relate to the elevated ASPS scores observed in Athletes B–D in the following, were screened for elevated 5α‐ADiol/A and 5β‐ADiol/Etio ratios, Ethyl glucuronide, 5α‐reductase inhibitor and ketoconazole concentrations, as well as the presence of heterodimeric hCG, anabolic steroids, aromatase inhibitors, masking agents and diuretics, and antiestrogens within the samples. The samples of Athletes B–D were not impacted by the aforementioned confounders.

The models underlying our risk scores have some limitations. First, we use the ABP scores to adjust for within‐subject correlation, which allows the use of statistical and machine learning classification methods. However, the ABP scores may not be able to remove all within‐subject correlation and therefore additional models, such as logistic mixed models, may be necessary if the correlation is substantial. Second, our approach uses both experimental data and doping control data. We allow for the difference in the informativeness of these types of data by a weighting scheme for the contributions to the log likelihood or loss. An alternative approach could be to allow for false positives from ADRVs within the doping control data and so, more naturally, adjust for the differences in the informativeness. However, this would lead to more computationally demanding inference schemes. It would also lose the ability to summarise the observed biomarkers using measures such as mean, variance and autocorrelation. Finally, our ability to determine risk scores is limited by the number of athletes with a sanction in the anti‐doping control data set.

## 5 Conclusions

This study documents the development of an Abnormal Steroid Profile Score (ASPS) to identify the probability of doping from changes in steroid biomarkers. The laboratory‐controlled sample data suggest the ASPS was able to discriminate between different doping methods and doses. Results from the analysis of doping control sample data indicate that the ASPS can also discriminate between those with and without an ADRV in a large data set of athletes. Our findings demonstrate that using sequence probability from the adaptive ABP model provides better AUC measures than using the original data for all methods. Moreover, analysing the trajectory of the whole steroidal profile provides better results, as it is possible to quantify the contribution of different sources (biomarkers or their interactions) to the ASPS. This can be used to more easily interpret athlete profiles and may also be useful to identify the steroids, which lead to a change in the ASPS, even if a mixture of steroids is used. Similar to the ABPS that has been used as an additional longitudinal marker of possible blood doping methods for years, these findings demonstrate the potential added value behind an ASPS that could further improve the focused assignment of costly IRMS analysis to identify anabolic agent doping and/or follow‐up sample collection when warranted.

## Funding

The authors have nothing to report.

## Conflicts of Interest

The authors declare no conflicts of interest.

## Data Availability

Research data are not shared.
